# A1M/α_1_-microglobulin is proteolytically activated by myeloperoxidase, binds its heme group and inhibits low density lipoprotein oxidation

**DOI:** 10.3389/fphys.2015.00011

**Published:** 2015-02-03

**Authors:** Martin Cederlund, Adnan Deronic, Jan Pallon, Ole E. Sørensen, Bo Åkerström

**Affiliations:** ^1^Division of Infection Medicine, Department of Clinical Sciences, Lund UniversityLund, Sweden; ^2^Division of Immunology, Department of Experimental Medicine, Lund UniversityLund, Sweden; ^3^Division of Nuclear Physics, Department of Physics, Lund UniversityLund, Sweden

**Keywords:** α_1_-microglobulin, myeloperoxidase, low density lipoprotein, C-terminal proteolysis, heme binding, neutrophils

## Abstract

α_1_-microglobulin (A1M) is a 26 kDa plasma and tissue protein with reductase activity and radical- and heme-binding anti-oxidative functions. In addition, exposure of A1M to hemoglobin has been shown to induce proteolytic elimination of a C-terminal tetrapeptide yielding a heme-degrading form, truncated A1M (t-A1M). Myeloperoxidase (MPO), a heme-containing enzyme that catalyzes the production of free radicals and hypochlorite, is released by neutrophils during the inflammatory response to bacterial infections. MPO-induced low density lipoprotein (LDL)-oxidation in blood has been suggested as a causative factor in atherosclerosis. In this study we have hypothesized that A1M interacts with MPO in a similar mode as with hemoglobin, and is a regulator of its activity. The results show that A1M is proteolytically cleaved, with formation of t-A1M, after exposure to MPO, and that t-A1M contains iron and heme-degradation products. The reaction is dependent of pH, time and concentration of substrates and a pH-value around 7 is shown to be optimal for cleavage. Furthermore, A1M inhibits MPO- and hydrogen peroxide-induced oxidation of LDL. The results suggest that A1M may have a role as an inhibitor of the damaging effects of the neutrophil respiratory burst on bystander tissue components.

## Introduction

The heme-containing enzyme myeloperoxidase (MPO) is a cationic dimer with a molecular mass of 146 kDa. The enzyme is stored in azurophil granules of non-stimulated neutrophils and participates in killing of microorganisms during phagocytosis (Nauseef and Malech, 1986; Malle et al., 2007). The enzyme catalyzes the conversion of hydrogen peroxide and chloride to the bactericidal compound hypochlorous acid/hypochlorite (Malle et al., 2007). MPO is a major component of the azurophil granules and is released into the extracellular compartment when de-granulation of the neutrophil is induced by various stimuli (Farschou and Borregaard, 2003). Hypochlorite and free radicals are important weapons against bacteria, but they also have the negative effect of oxidizing by-stander tissue components (Winrow et al., 1993; Klebanoff, 2005). MPO and the reactive intermediates it generates have been identified in atherosclerotic lesions and MPO may thus contribute to the oxidation of low density lipoproteins (LDL) that ultimately leads to atherosclerosis (Daugherty et al., 1994).

α_1_-microglobulin (A1M), also called protein HC, a plasma and tissue protein with antioxidative properties, was first discovered in human urine about 40 years ago (Ekström et al., 1975). It has a mass of 26 kDa, consists of 183 amino acid residues, and has been identified in a wide range of other species (Åkerström and Gram, 2014). A1M is a member of the lipocalin family of structurally related proteins in bacteria, plants and animals (Åkerström et al., 2006). The recently published crystal structure shows that the protein consists of an eight-stranded, anti-parallel β-barrel with a highly conserved cysteine residue in position 34 located in a loop near the open top of the beta-barrel (Meining and Skerra, 2012). The lipocalin pocket inside the β-barrel serves as a ligand-binding entity in many of the lipocalins (Flower, 1996). A1M is synthesized and secreted from liver and most other epithelial cells, and present in blood and all tissues at remarkably constant concentrations, 1–2 μM (DeMars et al., 1989). The protein is found in blood and tissues both in free form and bound to immunoglobulin A, albumin or prothrombin (Berggård et al., 1997).

A1M has been suggested to have a physiological function as a cell and tissue protective antioxidant that operates by clearing extravascular fluids of free radicals and heme groups (reviewed in Olsson et al., 2012; Åkerström and Gram, 2014). To achieve this, A1M employs enzymatic reductase, radical-scavenging, and heme-binding properties (Allhorn et al., 2002, 2005; Larsson et al., 2004; Åkerström et al., 2007). Several recent publications demonstrate that A1M indeed has powerful protective effects *in vivo* and *in vitro* against oxidative damage from free radicals, extracellular hemoglobin and free heme groups released during hemolysis (Olsson et al., 2008, 2011; May et al., 2011; Rutardottir et al., 2013; Rosenlöf et al., 2014; Sverrisson et al., 2014).

Hemolysis and release of free hemoglobin occurring in different pathological states results in generation of reactive oxygen species (ROS) by autooxidation of the oxy-hemoglobin molecule, and in increased levels of free heme (Olsson et al., 2012). Free heme and ROS contributes to oxidation, covalent cross-linking and aggregation of proteins, induce oxidative DNA-lesions, and catalyze the formation of cytotoxic lipid peroxidation (Kumar and Bandyopadhyay, 2005). Heme has been shown to be a pro-inflammatory molecule in pathological conditions like renal failure, atherosclerosis and heart transplant failure (Kumar and Bandyopadhyay, 2005). The heme-binding by A1M thus contributes to its antioxidative protective effects. The ability of A1M to bind heme has also been observed in four other mammals and fish (Larsson et al., 2004). When exposed to purified hemoglobin or lysed erythrocytes, a truncated A1M (t-A1M) is formed, lacking the C-terminal tetrapeptide LIPR (Allhorn et al., 2002). The presence of t-A1M in human urine indicates that this reaction occurs *in vivo*. T-A1M binds heme similar to full-length A1M but also shows a time-dependent spectral rearrangement indicating degradation of heme and formation of a heterogeneous chromophore.

In this work, we hypothesized that A1M, due to its heme-binding and reductive biochemical properties, can protect bystanding cells and molecules from oxidative damage by MPO. To approach this question, we have investigated the reactions occurring between MPO and A1M and the effects of A1M on the oxidation of LDL by MPO.

## Materials and methods

### Proteins and reagents

Recombinant human A1M with an N-terminal His_8_-tag was expressed in *E. coli*, purified and refolded as described by Kwasek et al. (2007), with the addition of ion-exchange chromatography performed as follows. The protein solution was applied to a column of DEAE-Sephadex A-50 (GE Healthcare, Uppsala, Sweden) equilibrated with the starting buffer (20 mM Tris-HCl, pH 8.0). A1M was eluted at a flow rate of 1 ml/min using a linear pH gradient consisting of 250 ml starting buffer and 250 ml elution buffer (20 mM Tris-HCl, 0.15 M NaCl, pH 8.0). Polyclonal rabbit antibodies against A1M were prepared by immunization with human urinary A1M as described by Berggård and Bearn (1968). Rabbit anti-LIPR antiserum was prepared by Agrisera AB (Vännäs, Sweden) by immunization with the synthetic peptide CKKLIPR conjugated to keyhole limpet hemocyanin (KLH). Goat anti-rabbit IgG was prepared, purified (Björck et al., 1977) and labeled with ^125^I using chloramin T as described (Greenwood et al., 1963). Bovine catalase was from Roche Diagnostics (catalog no. 106810; Mannheim, Germany). Purified neutrophil MPO was a kind gift from professor Inge Olsson, Department of Haematology, Lund University. Purity was assessed by SDS-PAGE as described below, and activity was estimated by EnzChek Myeloperoxidase (MPO) Activity Assay Kit (catalog no. E33856; Invitrogen, Eugene, OR, USA). Heme (ferriprotoporphyrin IX chloride) was purchased from Porphyrin Products, Inc. (Logan, UT, USA).

### Analysis of A1M reaction with heme and MPO

A1M, 1.2 mg/ml, was mixed with MPO, 1.2 mg/ml, or heme, 0.4 mM, in incubation buffer (25 mM sodium phosphate, pH 8) and incubated at room temperature for 1 or 24 h. The samples were analyzed by 12% SDS-PAGE with mercaptoethanol and 4–20% non-denaturing PAGE as described below.

To study cleavage efficiency, A1M (0.195 mg/ml) was mixed with different concentrations of MPO (0, 0.3, and 1 mg/ml) in incubation buffer. The samples were incubated at 37°C for 1, 4, and 24 h. After incubation, each sample was run on a 10% SDS-PAGE under reducing conditions. The same procedure was performed with another heme-containing control protein, catalase, instead of MPO. Samples with 0.195 mg/ml A1M and 0.1 mg/ml MPO were also mixed in buffers of different pH (0.3 M NaAc, pH 4; 0.3 M NaAc, pH 5; 0.3 M NaPi, pH 6; 0.3 M NaPi, pH 7; 0.3 M NaPi, pH 8; 0.3 M glycine-OH, pH 9), incubated at 37°C for 1 h and then run on a 12% SDS-PAGE under reducing conditions. The amounts of non-cleaved and cleaved A1M were estimated by densitometry of the bands using ImageJ 1.47v software (National Institutes of Health, USA).

### Electrophoresis and western blotting

SDS-PAGE was performed using the protocol of Laemmli (1970). Samples were mixed with sample buffer containing 2% (v/v) mercaptoethanol, boiled for approximately 1 min and applied to 12% linear, or 4–20% gradient polyacrylamide Criterion™ TGX™ Precast Gels (BioRad). Gels were stained with Coomassie Brilliant Blue R-250 (BDH Chemicals, Ltd. Poole, UK). High-molecular-mass standards (Rainbow markers, Amersham Pharmacia Biotech, Uppsala, Sweden) were used. Native PAGE was performed with no addition of SDS or mercaptoethanol in buffers and no boiling of samples. Following electrophoresis, the proteins were electrophoretically transferred to a PVDF membrane (Immobilon-P, Millipore, Bedford, MA, USA) as originally described by Matsudaira (1987), using Trans-Blot Turbo system from Bio-Rad. The membranes were blocked, incubated with specific rabbit antisera, followed by washing and incubation with Alexa Fluor 647 goat anti-rabbit IgG (dil 5000x; Molecular Probes). The membranes were developed in a ChemiDoc MP Imaging system (BioRad).

### Protein identification by mass spectrometry peptide mapping and sequencing analysis

The protein analysis was performed by Alphalyse A/S (Odense, Denmark). Briefly, the protein samples were reduced and carbamidomethylated, and subsequently digested with trypsin. The resulting peptides were concentrated on a ZipTip micropurification column and eluted onto an anchorchip target for analysis on a Bruker Autoflex Speed MALDI TOF/TOF instrument. MALDI MS/MS was performed on 15 peptides for partial sequencing. The MS and MS/MS spectra were combined and used for database searching. The data were searched against in-house protein databases downloaded from NBCI, including the NRDB database.

### Ion exchange chromatography of A1M/MPO samples

A 100 μl sample with A1M and MPO, both 1.2 mg/ml in incubation buffer, was incubated for 48 h at room temperature, and then analyzed by ion exchange chromatography, using an FPLC system (ÄKTAprime plus, GE Healthcare, UK) with a Mono-Q column washed with 20 mM Tris-HCl, pH 8. The proteins were eluted with 20 mM Tris-HCl, pH 8 by a 0–100% 0.5 M NaCl-gradient and absorbance was measured at wavelengths of 280 and 405 nm. The same procedure was performed with pure A1M and MPO separately in the same amounts. Fractions corresponding to the observed absorbance peaks were separated by 12% SDS-PAGE with mercaptoethanol. Fractions containing A1M that had reacted with MPO were analyzed by absorbance scanning between 250 and 750 nm on a Beckman (Beckman Instruments, Fullerton, CA) DU 800 spectrophotometer using a scan rate of 1200 nm/min. Absorbance scanning was also performed on pure A1M and MPO for comparison.

### Proton-induced X-ray emission (PIXE) analysis

Samples for proton-induced X-ray emission analysis were prepared as follows. Chromatography fractions in 20 mM Tris-HCl, pH 8.0 + 0.1–0.2 M NaCl, containing A1M in concentrations determined by UV-absorbance, were added to a Kimfoil membrane (Kimberley Clark) mounted on a plastic frame and allowed to dry. At the Lund Nuclear Microprobe facility (Malmqvist et al., 1993), the samples were placed in a vacuum irradiation chamber and bombarded with a 1 nA proton beam, with an energy of 2.55 MeV and accumulating a beam charge of 0.6 μC. The characteristic X-rays emitted (Johansson and Campbell, 1988) were detected using a KETEK AXAS-M H50 SDD Silicon Drift X-ray detector, while data were collected using KMAX (Sparrow) software (Elfman et al., 1997). Elemental standards (Fe) were analyzed in the same way to verify quantification.

### Neutrophil granulocyte isolation and cultivation

Buffy coat from a healthy donor was mixed with an equal volume of 2% Dextran in 0.9% NaCl and erythrocytes were allowed to sediment. The supernatant was aspirated and centrifuged at 200 g for 10 min at 4°C and the resulting pellet resuspended in 0.9% NaCl. The resuspension was layered on top of half the volume of Lymphoprep, centrifuged at 400 g for 25 min and the supernatant removed. Erythrocytes were lysed by addition of ice-cold H_2_O and the neutrophils were washed once with 1.8% NaCl centrifuged at 200 g for 6 min and washed twice with 0.9% NaCl. The cells were resuspended in Krebs Ringer glucose-buffer (10^6^ cells/ml) and cultured for 20 h at 37°C with or without addition of 1 μM ionomycin and 0.1 mg/ml A1M. The cells were spun down and the supernatants analyzed by 12% SDS-PAGE with mercaptoethanol and Western blotting with anti-A1M antibodies as described above. Supernatants from overnight cultures were also mixed with A1M (0.1 mg/ml) and/or PMSF (1 mM), after centrifugation and separation of the cells, followed by SDS-PAGE and Western blotting.

### Low density lipoprotein oxidation inhibition and reduction

Oxidation inhibition and reduction of oxidized LDL was measured by the thiobarbituric acid (TBA) method described by Gutteridge (1977). (1) Oxidation: 0.2 mg/ml LDL were incubated with 8 mM H_2_O_2_ and a dilution series of MPO (25–100 μg/ml) in a total volume of 200 μl PBS at 24°C for 2 h. (2) Inhibition: 0.2 mg/ml LDL were incubated with 8 mM H_2_O_2_, 100 μg/ml MPO or albumin and A1M (8, 20 μM) in a total volume of 200 μl PBS at 24°C for 2 h. 3) Reduction: 0.2 mg/ml LDL were incubated with 8 mM H_2_O_2_, 100 μg/ml MPO in PBS at 24°C for 2 h, then different dilutions of A1M (1–10 μM) were added in a total volume of 200 μl PBS and the samples were incubated at 24°C for 1 h. All samples (200 μl/sample) were mixed with TBA-reagent (0.5% 2-thiobarbituric acid and 0.5% SDS in H_2_O), 0.25 ml, and 0.2 M glycine-HCl, pH 3.6, 0.29 ml (Girotti and Thomas, 1984), was then added and the mixture heated to 95°C for 15 min. The absorbance at 550 nm was read after cooling to room temperature and centrifugation 3000 rpm/15 min. Origin 9.0 software (OriginLab Corporation, Northampton, MA, USA) was used to analyze the data.

## Results

### Formation of t-A1M by reaction with MPO

**Figure 1:**
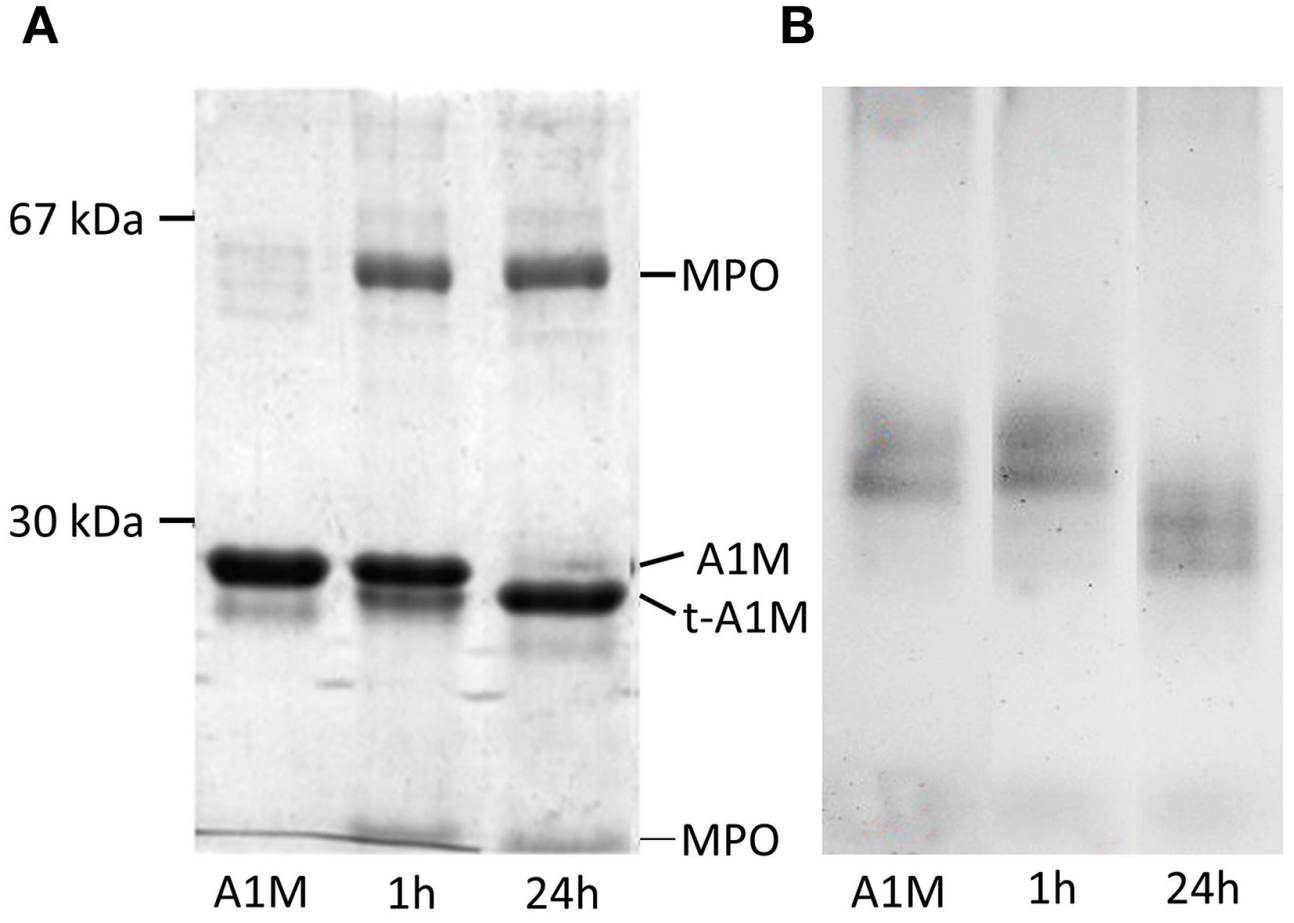
**MPO-induced proteolytic cleavage of A1M**. Lane 1 contains 3 μg A1M and lane 2 and 3 both contain 3 μg A1M mixed with 3 μg MPO in incubation buffer, incubated for 1 and 24 h respectively. **(A)** SDS-PAGE with mercaptoethanol. **(B)** Non-denaturing PAGE without mercaptoethanol.

A1M and MPO were mixed at the molar ratio 6:1, incubated for 1 and 24 h and analyzed by SDS-PAGE under reducing conditions. As can be seen in Figure 1A, a faster-migrating, truncated form of A1M was seen after interaction with MPO, indicating a proteolytic cleavage. The truncated form was similar in size to t-A1M previously shown to be formed by interaction with hemoglobin (Allhorn et al., 2002). The MPO-molecule was seen as two bands corresponding to the two subunit chains of the protein (Nauseef and Malech, 1986). A larger proportion of A1M was cleaved in the long-duration reaction (24 h), compared to the short-duration reaction (1 h), suggesting a time dependent activity. This difference in cleavage was confirmed by the presence of two bands in the lane with A1M from the 1 h-reaction, where all A1M still had not reacted with MPO. SDS-PAGE under non-reducing conditions of the samples yielded similar results (not shown). Non-denaturing PAGE was run on the same samples (Figure 1B) and shows separation based on both size and the net-charges of MPO and A1M. Two A1M-bands were seen, probably representing monomer and dimer. A clear increase of the net negative charge and/or decrease in size of A1M was seen after incubation with MPO as compared to A1M alone. Incubation of A1M with heme also yielded a third band with slower migration and thus decreased net negative charge (not shown), probably as a result of attachment of porphyrin and its iron to A1M.

**Figure 2:**
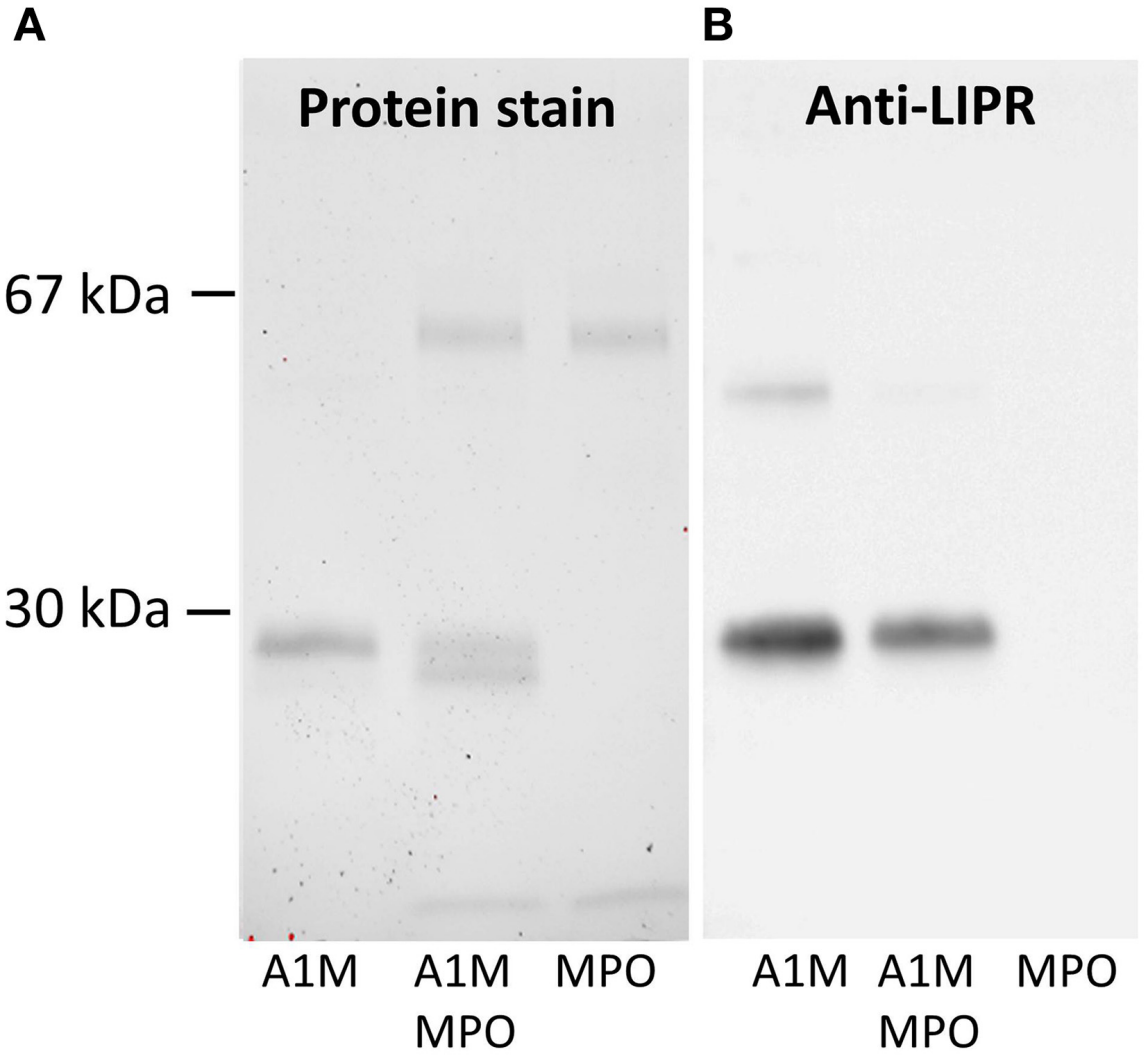
**Western blotting of A1M before and after MPO-induced cleavage**. The samples, 0.1 μg of full-length A1M, MPO and a mixture of A1M/MPO, 0.1 μg of each, incubated for 4 h, were separated on a 12% SDS-PAGE prior to transfer to a PVDF-membrane and blotting. **(A)** Staining with Coomassie. **(B)** Immunoblot with anti-LIPR antibodies.

To determine if the cleavage product is t-A1M, defined as A1M without its C-terminal tetrapeptide LIPR (Allhorn et al., 2002), a mixture of the two proteins with the molar ratio 1:6 (MPO:A1M) was incubated for 4 h. The cleavage products and non-cleaved A1M were analyzed by SDS-PAGE and analyzed for total protein (Figure 2A) and by immunoblotting using anti-LIPR (Figure 2B) antibodies. As expected, both forms of A1M were detected after Coomassie staining, while the anti-LIPR antibody only bound to full-length A1M. Finally, the two bands, i.e., cleaved and non-cleaved A1M-bands were analyzed by trypsin cleavage and mass-spectrometry after excision from the gel, confirming that LIPR was missing in the smaller band. These results demonstrated that A1M was cleaved by MPO in a similar reaction as with hemoglobin (Allhorn et al., 2002).

### A1M cleavage is dependent on concentration, time and PH

**Figure 3:**
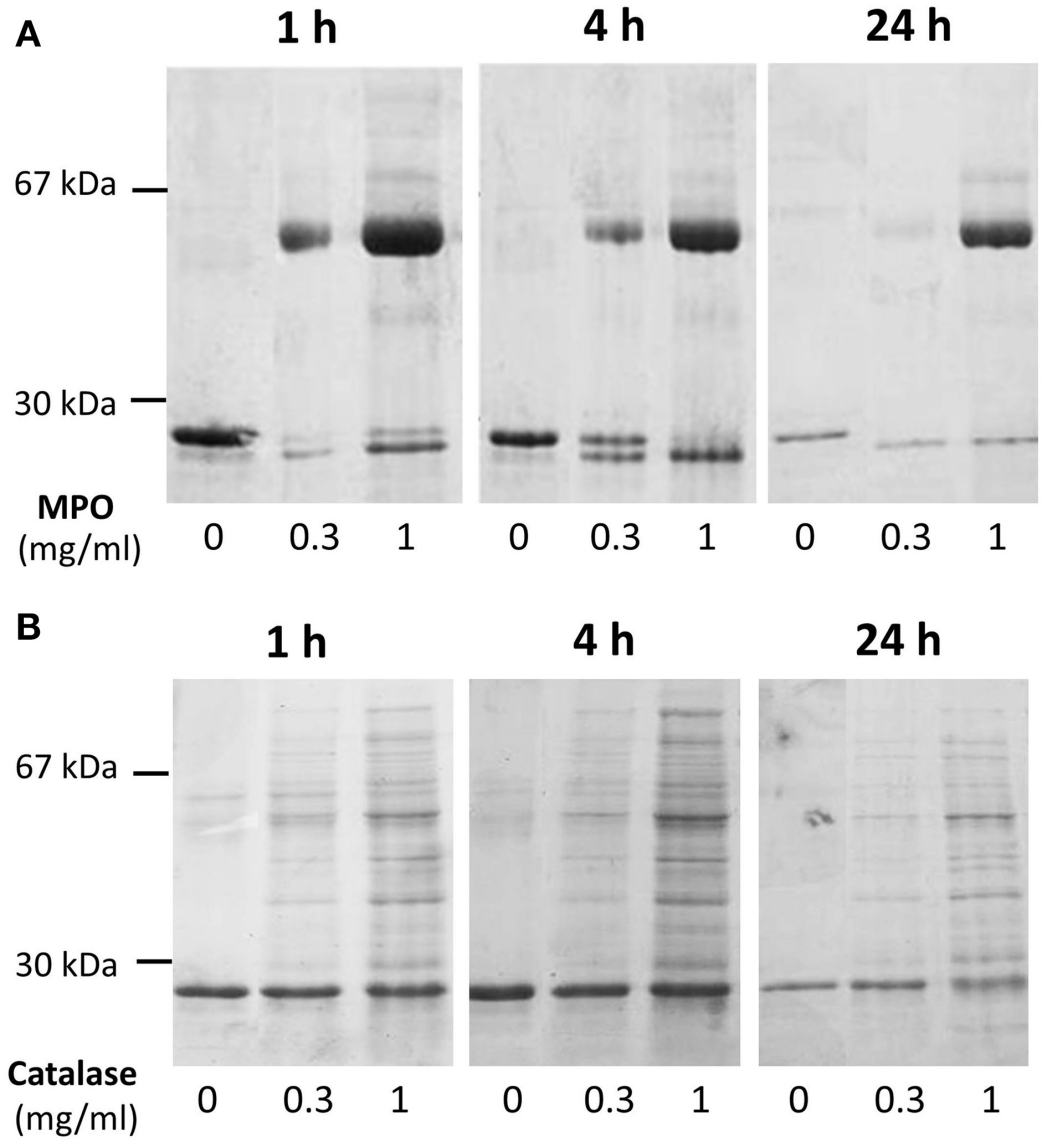
**Time- and concentration dependence of MPO-induced cleavage of A1M**. **(A)** A1M (0.195 mg/ml) was incubated with MPO at concentrations of 0, 0.3, or 1 mg/ml for 1, 4, or 24 h at room-temperature. Ten microliters of the samples were separated on a 12% SDS-PAGE gel in the presence of mercaptoethanol, and then stained with Coomassie. One representative experiment is shown. **(B)** A similar experiment with bovine catalase under the same conditions was performed.

**Figure 4:**
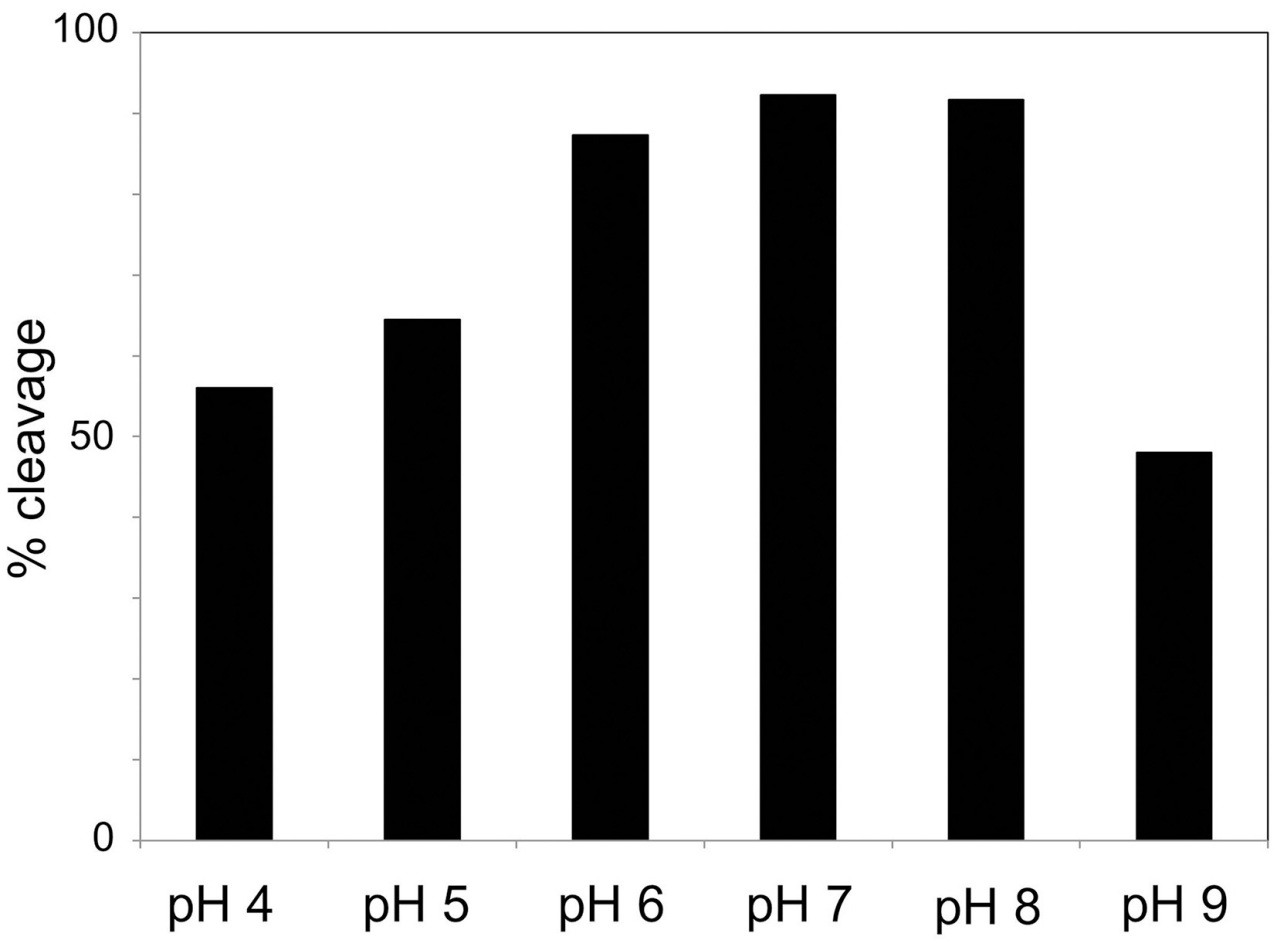
**pH-dependence of MPO-induced cleavage of A1M**. A1M was mixed with MPO at a concentration of 0.195 mg/ml and 0.1 mg/ml respectively, in buffers with different pH. The samples were incubated for 1 h at 37°C. Ten microliters of the samples were then separated by SDS-PAGE on 12% gels in the presence of mercaptoethanol. The gels were stained with Coomassie and quantified by densitometry as described in Materials and Methods. One representative experiment is shown.

Cleavage efficiency was measured in a mix of A1M at 0.195 mg/ml with different concentrations of MPO (0 mg/ml, 0.3 mg/ml, and 1 mg/ml), and different incubation times (1, 4, and 24 h). The results after SDS-PAGE (Figure 3A) showed increasing cleavage of A1M with higher concentrations of MPO and longer incubation times. When comparing the lanes containing MPO at 1 mg/ml, bands for both truncated and full-length A1M was clearly seen in the gel with samples incubated for 1 h (Figure 3A). The cleavage was also time-dependent, i.e., more A1M was cleaved with time (4 and 24 h; Figure 3A) and after 24 h only the t-A1M band was seen, suggesting that all A1M had reacted with MPO. A faint t-A1M band was seen with no MPO present. This could be explained by some cleavage of A1M in the *E. coli* where A1M was expressed. As a control, another heme-containing protein, catalase, was incubated with A1M under similar conditions (Figure 3B). This did not yield any cleavage of A1M as seen by SDS-PAGE. The efficiency of cleavage was also tested at different pH-values (Figure 4) using 1 h incubation time and a molar ratio of A1M and MPO of 1:3. The mixtures were separated by SDS-PAGE and the relative amounts of the two bands were estimated by densitometry after Coomassie staining. As can be seen from Figure 4, most A1M cleavage occurred in the pH range 6–8 indicating that A1M activity is most efficient at physiological pH. A lower degree of cleavage was observed at pH 4, 5, and 9.

### Heme group of MPO is transferred to A1M

**Figure 5:**
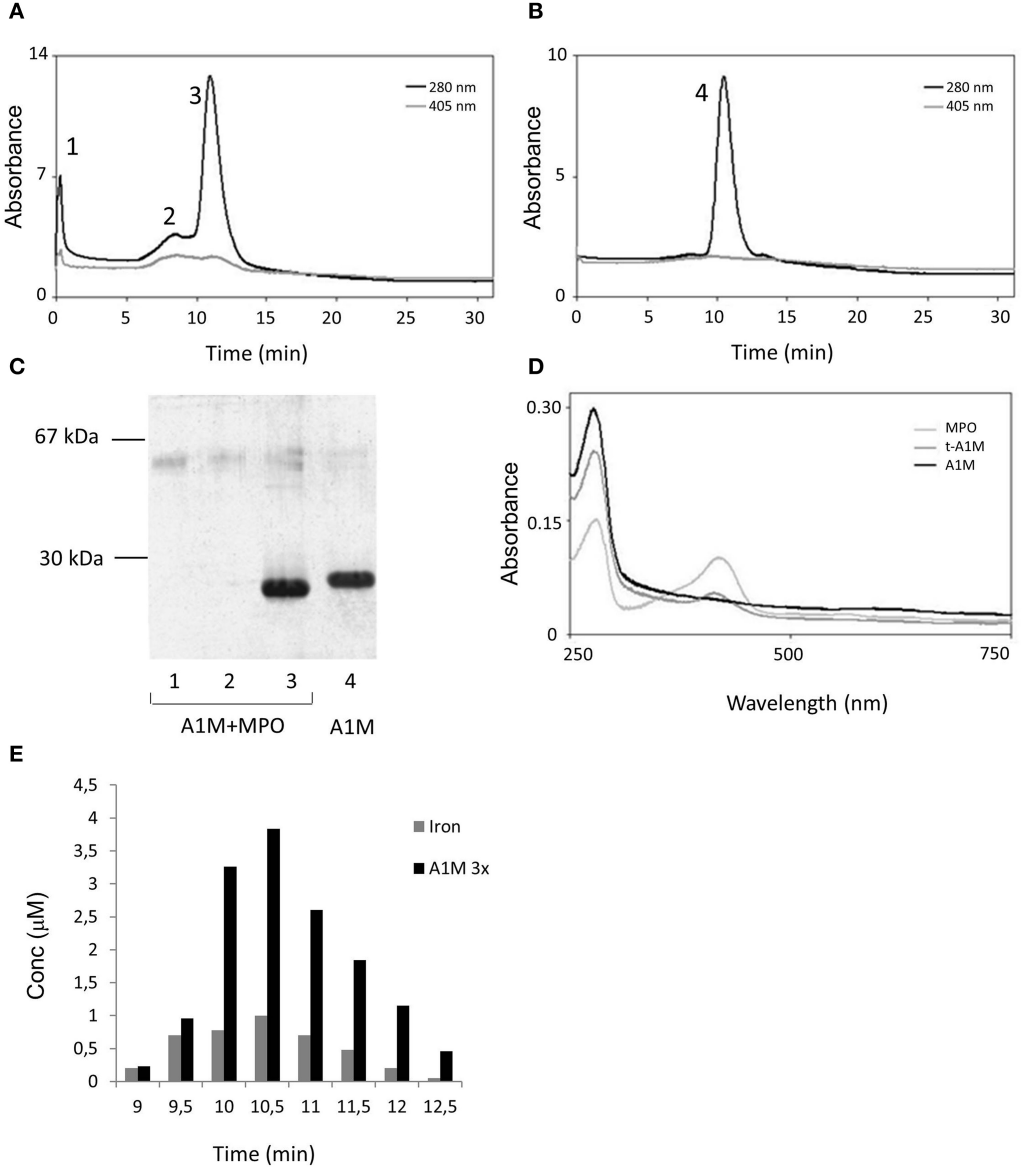
**Ion exchange chromatography of an A1M/MPO mixture**. **(A)** A1M and MPO (mol:mol, 6:1) were incubated at room temperature for 48 h and applied to a Mono-Q column. The column was eluted with a 0–0.5 M sodium chloride gradient in 20 mM Tris-HCl, pH 8. Fractions of 0.5 ml were collected and absorbance at 280 and 405 nm measured. **(B)** As a comparison, the same procedure was done on a sample with pure A1M. **(C)** SDS-PAGE analysis of the peak-fractions, marked 1–4 in **(A,B)**, obtained after ion exchange chromatography of the A1M/MPO mix and the pure A1M sample. **(D)** Absorbance scanning between 250 and 750 nm of fractions obtained after ion exchange chromatography of the A1M/MPO mix shown to contain t-A1M by SDS-PAGE in **(C)** (lane 3). As a comparison, the same procedure was done on a sample with pure A1M and MPO. **(E)** Size-exclusion chromatography on Superose 12 of a mixture of A1M and MPO (molar ratio 6:1), incubated at room-temperature for 48 h, followed by analysis of A1M (absorbance and SDS-PAGE) and iron content measured by PIXE in selected fractions. The A1M-concentrations are given after dilution three times, for optimal illustration.

To analyze the reaction products in more detail, A1M and MPO were incubated at pH 8 for 48 h at the molar ratio 6:1 and then separated by ion exchange chromatography, eluting the proteins by a salt concentration gradient. As a control, A1M and MPO alone were subjected to the same procedure (not shown). The eluates were analyzed by absorbance at 280 and 405 nm (Figures 5A,B) and by SDS-PAGE (Figure 5C). The A1M/MPO mix was eluted as three peaks and, as shown by SDS-PAGE, the first two peaks contained MPO and the third peak contained t-A1M. Pure A1M is eluted at a slightly lower salt concentration than A1M that had reacted with MPO, which again indicated an increase in net negative charge. Absorbance scanning showed a maximum at 280 nm in all samples (Figure 5D), and another absorbance maximum, at 410–420 nm, was observed in samples with pure MPO and A1M that had reacted with MPO, but not with pure A1M. This maximum, a Soret band, is characteristic for the heme group (Allhorn et al., 2002), and indicates the presence of heme/porphyrins in MPO as well as in A1M derived from the mixture. The absorbance spectra suggested that the heme/porphyrin group was transferred from MPO to A1M when A1M reacts with MPO.

Iron analysis of the fractions from the ion-exchange chromatography showed the presence of iron in the peak A1M-fraction at a molar ratio of 1:4 (Fe:A1M; not shown). The A1M+MPO mixture was also separated by gel chromatography on Superose 12 followed by iron analysis of the fractions (Figure 5E). Also in this case, the A1M-containing fractions showed correlated iron and A1M concentrations, and the A1M-peak fractions show a molar ratio of 1:10 (Fe:A1M). These results suggest that the heme-group bound by A1M partially contains the iron-atom.

### A1M is cleaved after incubation in neutrophil medium

**Figure 6:**
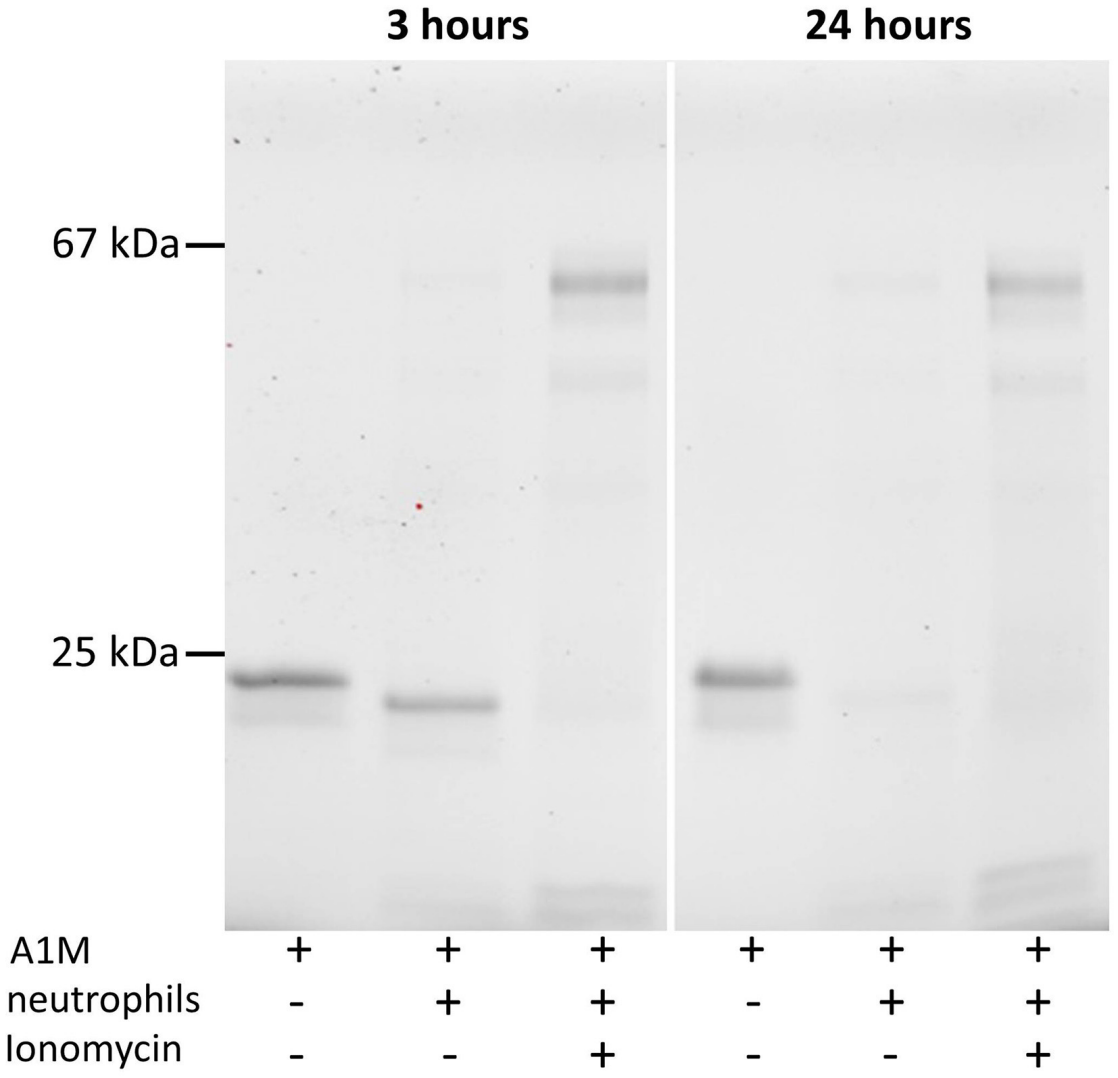
**Cleavage of A1M in neutrophil culture medium**. SDS-PAGE on 12% gels in the presence of mercaptoethanol followed by Western blotting with anti-A1M antibodies. The left lanes show A1M (0.1 mg/ml) incubated in fresh medium, the middle lanes show A1M incubated with neutrophil culture medium (0.1 mg/ml), and the right lanes show A1M (0.1 mg/ml) incubated with culture medium together with neutrophils activated by ionomycin. Culture conditions are described in Materials and Methods. Left panel, incubation for 3 h, and right panel shows the same procedure but with 24 h incubation.

Neutrophils extracted from buffy coat of a healthy donor were mixed with 0.1 mg/ml A1M in PBS in order to determine whether A1M would be cleaved by cellular MPO released into the culture medium. Ionomycin, 1 μM, was also added to some samples to induce degranulation. SDS-PAGE of the medium after 3 and 24 h (Figure 6) shows that t-A1M is formed in the medium, and the cleavage was complete after 3 h, even without ionomycin-induced degranulation. After degranulation, the presence of MPO in the medium can be seen as two bands, and was confirmed by immunoblot analysis using anti-MPO antibodies (not shown). T-A1M is also seen after ionomycin-induced degranulation, but at lower concentrations, suggesting that proteolytic degradation of the protein occurs. After 24 h, smaller amounts of t-A1M is seen with or without ionomycin-addition, again indicating proteolytic degradation. Addition of A1M to the cell supernatants alone, i.e., after centrifugation and removal of the cells, also resulted in the formation of t-A1M (not shown). Addition of the serine protease inhibitor PMSF, 1 mM, in order to reduce possible interactions between A1M and the potent neutrophil serine proteases did not prevent the cleavage of A1M.

### A1M inhibits LDL oxidation by MPO

**Figure 7:**
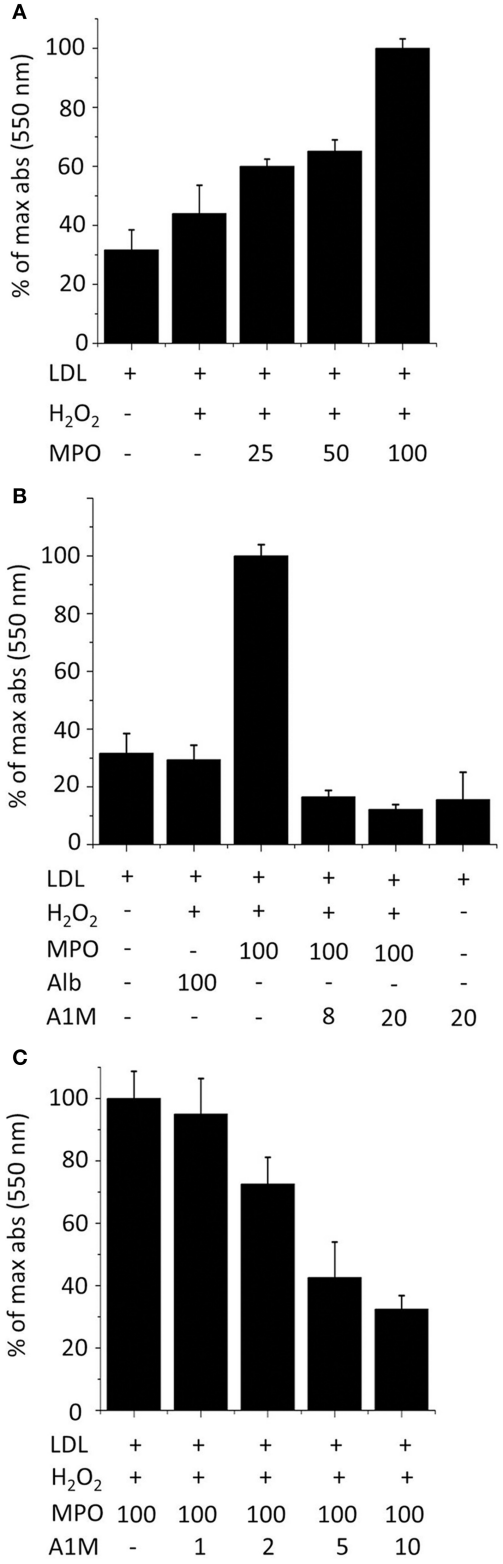
**A1M inhibits MPO-induced oxidation of LDL**. **(A)** LDL, 0.2 mg/ml, was incubated with 8 mM H_2_O_2_ and a dilution series of MPO (25–100 μg/ml) in a total volume of 200 μl PBS at 24°C for 2 h followed by TBARS analysis. **(B)** LDL, 0.2 mg/ml, was incubated with 8 mM H_2_O_2_, 100 μg/ml MPO and albumin or A1M (8, 20 μM) in a total volume of 200 μl PBS at 24°C for 2 h followed by TBARS analysis. **(C)** LDL, 0.2 mg/ml, was incubated with 8 mM H_2_O_2_, 100 μg/ml MPO in PBS at 24°C. After 2 h, a dilution series of A1M (1–10 μM) was added in a total volume of 200 μl PBS, and the samples were incubated at 24°C for 1 h followed by TBARS analysis. Results are from triplicate experiments and presented as mean ± SD.

LDL, in the presence of H_2_O_2_, was oxidized in a dose dependent fashion by MPO after 2 h incubation (Figure 7A), as measured by the TBA assay. The oxidation was inhibited by A1M added to the reaction mixture (Figure 7B). The TBA-signal was reduced to below the values of non-oxidized LDL by the addition of A1M, and A1M alone also had some reducing effects on non-oxidized LDL. To analyze whether A1M also had any effect on LDL after the oxidation-reaction had occurred, the protein was added only after 2 h preincubation of MPO with LDL. As seen in Figure 7C, a dose-dependent reduction of the TBA-signal was seen, suggesting that A1M was able to reduce the pre-oxidized LDL.

## Discussion

The results of this study show that A1M undergoes a similar reaction with MPO as previously shown with hemoglobin (Allhorn et al., 2002). The reaction resulted in proteolytic cleavage of A1M, loss of the C-terminal tetrapeptide, LIPR, and formation of a truncated form of A1M, called t-A1M. Catalase, another heme-containing protein, did not induce formation of t-A1M, suggesting that the cleavage reaction is specific for hemoglobin and MPO.

The absorbance spectrum of t-A1M after the reaction with MPO suggests that the heme group is transferred from MPO to A1M. Also in this respect, the reaction is similar to the reaction between A1M and hemoglobin (Allhorn et al., 2002). Analysis of the cleaved A1M after purification from MPO suggests that iron is still bound to the porphyrin, but at a lower than 1:1 molar ratio. This is supported by the presence of a Soret band in t-A1M, which is an indicator of chelated iron. In spite of this, the net negative charge of t-A1M was increased, as shown by native PAGE as well as ion exchange chromatography. Part of this increase could be explained by a loss of the positively charged C-terminal tetrapeptide LIPR, but it is also possible that other modifications of A1M are induced by the reaction with MPO. A1M has previously been shown to bind heme-groups in the molar ratio 1:2 (A1M:heme) (Siebel et al., 2012), and analysis by native PAGE showed that the A1M:heme complex had an increased net negative charge (Karnaukhova et al., 2014). *In vivo*, the lysyl side groups in positions 92, 118, and 130 are covalently modified on A1M from plasma and urine, and the modified protein is brown-colored and has increased negative charge and charge-heterogeneity (Calero et al., 1996; Berggård et al., 1999; Sala et al., 2004). Thus, heterogeneous modifications of these lysine residues as a result of the reaction with the heme-group could possibly explain the increased net negative charge after binding the heme-group.

In contrast to hemoglobin, the heme-group in MPO is covalently bound to the protein by two ester linkages as well as a sulfonium ion linkage (Fiedler et al., 2000), suggesting that a different mechanism is involved when heme is transferred from MPO to A1M compared to the heme-transfer from hemoglobin. It can be speculated, however, that similar heme degrading mechanisms are involved once the heme-group has been incorporated to the A1M protein.

Cleavage of A1M was also observed after incubation with cultured neutrophil granulocytes (Figure 6). The formation of t-A1M in the culture medium could be verified using anti-LIPR antibodies (not shown). Addition of ionomycin to the neutrophils to induce azurophil degranulation and release of MPO as well as proteolytic enzymes resulted in large amounts of MPO in the culture medium. However, the concentration of t-A1M was lower after degranulation, indicating that non-specific proteolytic degradation of A1M was taking place after degranulation. Likewise, longer incubation times, 24 h, also resulted in proteolytic loss of the A1M protein. Taken together, the results suggest that A1M can be proteolytically activated by non-degranulated neutrophils. *In vivo*, t-A1M has been detected in several different physiological tissue fluids, including blood, urine and extravascular fluids (Lopez et al., 1982; Allhorn et al., 2002, 2003). It has been speculated that t-A1M results from proteolytic cleavage of full-length A1M induced by free hemoglobin in the tissues, originating from hemolysis. The results in this paper indicate that neutrophil-derived MPO also may participate in the formation of t-A1M *in vivo*.

For example, t-A1M is known to be present in inflamed tissue in chronic ulcers (Allhorn et al., 2003). Its role in this context is unknown but the inhibition of MPO-induced oxidation of LDL, as shown in Figure 7, suggests that it may be involved in down-regulating the oxidative stress of hypochlorite and free radicals, synthesized by MPO during the respiratory burst (Klebanoff, 2005). Therefore, A1M may be part of a defense mechanism protecting bystander tissues from oxidative damage by the oxidative burst of neutrophils. Such a novel down-regulatory mechanism of inflammation may play a role in a wide range of diseases, such as atherosclerosis, multiple sclerosis, rheumatoid arthritis, etc.

**Figure 8:**
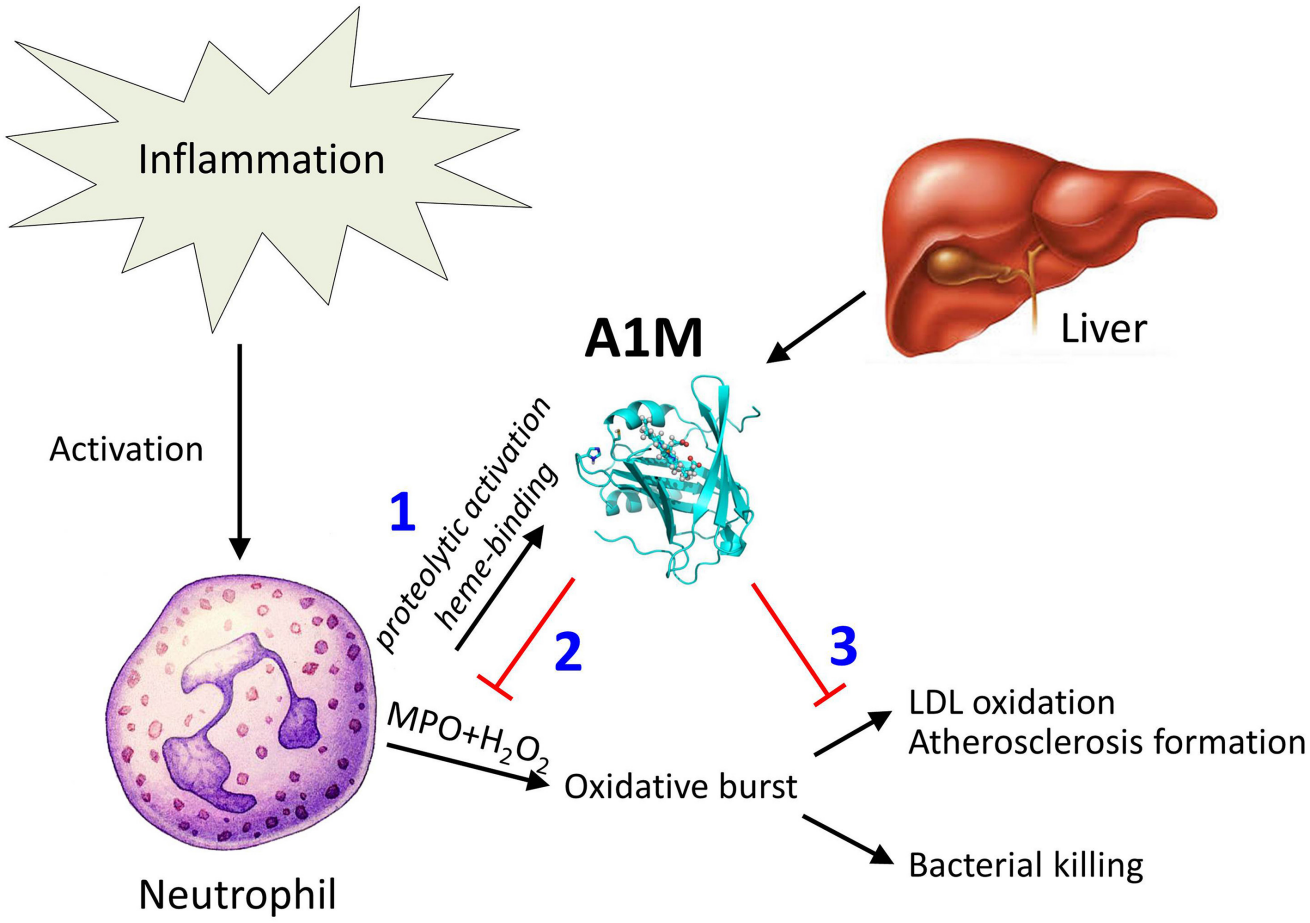
**Tentative function of A1M in protection against bystander oxidative damage caused by neutrophils**. The figure summarizes the findings of this paper in a physiological context. Recruitment and activation of neutrophils during inflammation involves MPO-mediated bacterial killing but may also yield LDL-oxidation as a side-effect. (1) Neutrophil culture medium or purified MPO induces heme-binding of A1M, and proteolytic cleavage of the protein which was previously shown to activate a heme-degradation activity of A1M (Allhorn et al., 2002). (2) The induced heme-degradation mechanism of A1M is tentatively “disarming” the peroxidase, preventing oxidative burst. (3) LDL-oxidation by MPO is inhibited by A1M, and pre-formed oxidation products on LDL are reduced.

Figure 8 summarizes a tentative physiological A1M-dependent mechanism for protection of LDL and bystander tissues against oxidation from neutrophil oxidative burst. Several potential mechanisms may explain how A1M achieves the protection of the LDL-particles. The cleavage of A1M and the binding and degradation of the heme-group indicates that A1M can disarm the MPO-enzyme by “stealing” its heme-group. Previous publications on A1M have shown that it also is a radical scavenger (Åkerström et al., 2007) and it reasonable to hypothesize that the protein also may operate by “scavenging,” or binding, the free radicals and hypochlorite generated by MPO. It was also shown in this work that oxidized or non-oxidized LDL-particles could be reduced by A1M even after the formation of oxidized modifications (Figure 7C). This is analogous to a similar effect of A1M on collagen fibrils: incubation of heme- or hydrogen peroxide-oxidized collagen with A1M resulted in a reduced amount of the oxidation product carbonyl groups (Olsson et al., 2011; Rutardottir et al., 2013). Mechanistically, this “cleaning” effect could be explained by the previously reported reductase activity of A1M (Allhorn et al., 2005), i.e., that A1M could chemically reduce the oxidation products in the macromolecules.

## Conclusion

MPO induces cleavage of LIPR from A1M in a time-, concentration- and pH-dependent manner which suggests that heme is transferred from MPO to A1M. A1M can also protect LDL against oxidative damage caused by MPO and may thus have an important role in preventing the formation of atherosclerosis. Further investigations are needed to clarify detailed mechanisms involved in the reactions between MPO and A1M, and may also reveal a correlation between A1M, MPO and patients with cardiovascular diseases.

### Conflict of interest statement

The authors declare that the research was conducted in the absence of any commercial or financial relationships that could be construed as a potential conflict of interest.

## Abbreviations

A1M: α_1_-microglobulin
t-A1M: truncated A1M
MPO: myeloperoxidase
LDL: low density lipoprotein
ROS: reactive oxygen species
protein HC: human complex-forming protein heterogeneous-in-charge
LIPR: leucine-isoleucine-proline-arginine
FPLC: fast flow liquid chromatography
PIXE: proton-induced X-ray emission
TBA: thiobarbituric acid
MS: mass spectrometry.
